# Burnout among general practitioners, a systematic quantitative review of the literature on determinants of burnout and their ecological value

**DOI:** 10.3389/fpsyg.2022.1064889

**Published:** 2022-12-15

**Authors:** Nicolaas Cornelis Verhoef, Robert Jan Blomme

**Affiliations:** ^1^Faculty of Management, Open University of the Netherlands, Heerlen, Netherlands; ^2^Faculty of Leadership and Management, Nyenrode Business University, Breukelen, Netherlands

**Keywords:** general practitioners, systematic quantitative literature review, occupation-specific determinants, generic determinants, ecological validity

## Abstract

Burnout is a major social and economic problem, specifically among general practitioners (GPs). The amount of literature on generic determinants of burnout is impressive. However, the size of the library on occupation-specific determinants of burnout among GPs are minimal. With the present study, we aim to gain insight into the existing academic literature on generic and occupation-specific determinants of burnout among GPs. Moreover, we aim to contribute to the ecological validity of this study by emphasizing occupation-specific determinants. We conducted a systematic quantitative literature review in which we followed the PRISMA statement and performed quality assessments according to the AXIS, CASP, MMAT, and 3-MIN procedures. Furthermore, we assessed frequency effect sizes (FES) and intensity effect sizes (IES). By performing Fisher’s exact tests, we investigated whether the quality of the studies influenced the outcomes. An extensive literature search revealed 60 eligible studies among which 28 strong studies, 29 moderate studies, and 3 weak studies were identified. Analyzing those studies delivered 75 determinants of burnout, of which 33 were occupation-specific for GPs. According to the average FES, occupation-specific determinants play a significant role in acquiring burnout compared to the generic determinants. The results of the Fisher exact tests provided evidence that the quality of the 60 studies did not affect the outcomes. We conclude that it is surprising that a profession with such an important social position and such a high risk of burnout has been so little researched.

## Introduction

In the general working population, burnout is considered a significant social and economic problem (Collier, 2017). In contrast to many other professions, relatively little is known about the causes of burnout among general practitioners (GPs).

Burnout is a psychological syndrome characterized by the three core symptoms of emotional exhaustion, depersonalization, and diminished personal effectiveness (Freudenberger and Richelson, 1980; Maslach et al., 2001). Since the introduction of the concept of burnout in the 1970s by Herbert Freudenberger, many studies have passed, attempting to explain this intriguing phenomenon (Freudenberger, 1974). Indeed, after introducing the Maslach Burnout Inventory (MBI) (Maslach and Jackson, 1981), a psychological assessment tool to measure the three dimensions of burnout, the research on this subject exploded (Maslach et al., 1996). Not surprisingly, considering the background of Freudenberger, the initial researchers found burnout exclusively related to human services, such as social work, healthcare, and teaching (Maslach and Schaufeli, 1993). However, the historical imperfection of exclusively relating burnout to human services, has been corrected abundantly in the past two decennia given a large amount of literature outside the human service sectors (Maslach and Schaufeli, 1993). Moreover, there was no theoretical argument to limit burnout to the human service professions (Maslach and Leiter, 1997). Moreover, there is enough evidence that shows that stressors leading to burnout in human services can be found in other non-human service professions as well (Buunk et al., 1998). Therefore, there appears to be sufficient theoretical arguments for releasing the initial conventional definition, restricting burnout to the human service professions. To meet this shortcoming of the Maslach Burnout Inventory-Human Services Survey (MBI-HSS), the Maslach Burnout Inventory–General Survey (MBI-GS) was developed, which was suitable for all workers, including those without intensive contact with recipients of services. One of the main differences between the two versions of the MBI was that the depersonalization scale from the MBI-HSS was transformed into the cynicism scale in the MBI-GS. Both scales reflect the social perception of distancing, distancing from people in the case of depersonalization and distancing from things in the case of cynicism (Schaufeli and Leiter, 1996). Simultaneously, the Oldenburg Burnout Inventory (OLBI) was developed, which consists of two dimensions: emotional exhaustion and disengagement from work (Demerouti, 1999). Despite their strengths and weaknesses, the MBI-HSS, the MBI-GS, and the OLBI are all widely practised.

According to Schaufeli and Taris (2005), burnout should be conceptualized, primarily as a work-related syndrome with at least emotional exhaustion and depersonalization as dimensions. One of the criticisms of the MBI is that depersonalization is a coping strategy under certain circumstances and should be better studied along with other coping strategies (Tipa et al., 2019). Furthermore, diminished personal efficiency, one of the three burnout dimensions of the MBI, should be perceived as one of the many consequences of long-term stress (Kristensen et al., 2005). These and other objections are amply and dignifiedly refuted by Schaufeli and Taris (2005). To address these concerns, the OLBI (Demerouti et al., 2003) was developed. One of the limitations of the OLBI is its limited construct validity (Halbesleben, 2003). However, a detailed discussion of this debate is beyond the scope of this study. Despite all the criticism, both the OLBI and the MBI are still widely used measurement tools today.

It is remarkable and as yet not easy to explain why the MBI is still so popular, despite all the criticism.

In response to the many well-documented objections to the MBI, a new Burnout Assessment Tool (BAT) has been developed (Schaufeli et al., 2019, 2020). The BAT comprises four core dimensions: exhaustion, mental distance, and emotional and cognitive impairment. Moreover, three secondary dimensions emerged: depressed mood, psychological distress, and psychosomatic complaints.

To classify the determinants of burnout globally, this study distinguished between job demands, job resources, and personal resources. Job demands are defined as those physical, social or organizational aspects of the job that require sustained physical or mental effort and are therefore associated with maintaining physiological and psychological costs (Demerouti et al., 2001). Examples of generic job demands are work overload and interpersonal conflict. Job resources were defined as those physical, social or organizational aspects of the job that may do any of the following: (a) be functional in achieving work goals; (b) reduce job demands and the associated physiological and psychological costs; (c) stimulate personal growth and development (Demerouti et al., 2001). Examples of generic job resources are feedback and social support. Personal resources are defined as the psychological characteristics or aspects of the self that are generally associated with resiliency and that refer to the ability to control and impact one’s environment successfully (Schaufeli and Taris, 2014). Examples of personal resources are self-efficacy and optimism. Extensive overviews of generic job demands, generic job resources and personal resources have been published by, among others, Lee and Ashforth (1996) and Schaufeli and Taris (2014).

Common theories in this regard are the job demands resources (JDR) theory and the conservation of resources (COR) theory. The JDR theory states that a balance between all possible work demands and all possible resources leads to the health and wellbeing of the worker (Demerouti et al., 2001; Schaufeli and Taris, 2014). The JDR model distinguishes two underlying processes, namely the health-limiting or energetic process and the motivational process (Demerouti et al., 2001). The health-limiting process arises at work demands and is mainly focused on exhaustion. The motivational process arises at work resources and is mainly focused on depersonalization. The JDR theory explains that there is an interaction between job demands and work resources, but the theory does not explain why, this requires additional psychological theories, such as the COR theory (Hobfoll, 2002). The central assumption of the COR theory is that people strive to preserve and protect the things they value, the resources. According to the COR theory, stress then arises when critical resources are threatened with loss, when they are lost, or when they are insufficiently regained after intensive effort (Hobfoll, 2002).

To empirically assess occupational stress, researchers usually study the effect of work demands and work resources on, for example, burnout. This research is usually done through questionnaires with generic job demands and job resources (Brough et al., 2009). The use of generic job demands and job resources is primarily driven by the wide range of occupations to which they apply so that the outcomes of studies can be compared between themselves but also with normative data (Brough et al., 2009).

The inclusion of occupation-specific job demands and job resources focuses on the assessment of the specific work context. This not only increases the ecological validity of the assessment but also improves the description of the work environment (e.g., Brough and Frame, 2004). The assessment of occupation-specific job demands and job resources is also beneficial for the development of targeted interventions (e.g., O’Driscoll et al., 2009). Finally, it is often overlooked by researchers that examining occupation-specific job demands and job resources simultaneously, yields additional explained variance over the generic job demands and job resources (e.g., Kop et al., 1999).

Occupational job demands and job resources have now been examined for various professions, for example, police officers (Juniper et al., 2010), teachers (Van der Doef and Maes, 2002; Bakker et al., 2007), firefighters (Tuckey and Hayward, 2011), prison staff (Brough and Biggs, 2015), nurses (Sundin et al., 2011), dentists (Hakanen et al., 2005), and GPs (Bakker et al., 2000a; Cathebras et al., 2004).

Traditionally, stressors or job demands of GPs have been related to the emotionally charged doctor-patient relationship. The demanding nature of the doctor-patient relationship has once been considered the root cause of burnout (Maslach, 1978). In the meantime, other than emotionally bound occupation-specific job demands for GPs have been identified, for example, high workload, and administrative job demands but also conflicts with social work (Cathebras et al., 2004).

Given the above-described added value of research into occupation-specific job demands and job resources, and the importance for a society of a well-functioning and healthy primary healthcare system, in particular for GPs, it is surprising that so little is known about occupation-specific job demands and job resources of GPs. The amount of academic literature on the determinants of burnout is impressive. However, the amount of literature on the determinants of burnout among GPs is much less. The amount of literature on occupation-specific determinants of burnout among GPs is very limited.

Therefore, the present study aims to gain insight into the existing academic literature on generic and occupation-specific determinants of burnout among GPs. We aim to contribute insight into and knowledge of the determinants that govern the mental energy household of GPs and that often lead to burnout. Moreover, by emphasizing occupation-specific determinants, we want to contribute to the ecological validity of this study.

We propose the following research question for the present study:

***What is the current state of knowledge regarding the generic and occupation-specific determinants of burnout among GPs?***

## Methods

In the present study, we used the systematic quantitative literature review (Pickering and Byrne, 2014). The method is systematic because of how studies are initially identified for inclusion, i.e., explicit and reproducible. The method quantifies where there is research but also where there are gaps. The technique can be used for all types of studies, qualitative, quantitative and mixed-method. The method bridges the gap between traditional narrative review methods and meta-analyses (Pickering and Byrne, 2014).

The present study was conducted in four phases. First, the literature search was conducted over the period 1970–2021. In the first phase, existing literature on determinants of burnout among GPs was collected. The beginning of the search marks a period when the term burnout was introduced in the scientific literature by Freudenberger (1974) and Maslach (1976). The search was performed with the search terms “burnout,” “general practice,” “GP,” “family practitioner,” “family doctor,” “family physician,” “primary care,” “primary care practitioner,” “primary care doctor,” “primary care physician,” and combinations of these search terms. The following search engines were used: Open University digital library interface, including Web of Science, Embase, PsycINFO, Google Scholar, PubMed, and Mendeley.

The following inclusion criteria were used in the second phase, the screening phase, to narrow the natural result. An abstract of an identified study must be available for screening purposes, and the language of the study must be Dutch or English. Type of study means any study that addresses the nature of a determinant and its relationship with burnout, i.e., no intervention studies. In 2016, a comparative study on GPs was published by Schäfer et al. (2016), which included 34 nationalities because they have comparable and well-documented primary care. It concerns 31 European countries, Canada, Australia, and New Zealand. The inclusion criterion “Nationality” in the present study is based on the said study by Schäfer et al. (2016), i.e., we used the 34 nationalities as a “Nationality” inclusion criterion. A separate inclusion criterion, the “Language” criterion, which is separate from the “Nationality” criterion, is the language in which the study is published and must be English or Dutch.

The USA is a rich source of scientific literature on primary care. This literature has provided evidence that a well-developed primary healthcare system offers benefits in terms of better coordination, continuity of care and possibilities for cost control. However, part of this research has limited relevance to the European situation (Kringos et al., 2010). Therefore, in the current study, we have limited ourselves to the European situation, and a comparable situation, i.e., the 34 nationalities mentioned in the study by Schäfer et al. (2016).

After identifying duplicate studies and screening on title and abstract with the above inclusion criteria, phase three followed.

In the third phase, the full text of the remaining studies was reviewed and assessed against the same inclusion criteria as in the previous screening phase. Naturally, the full text of the study had to be available. The remaining studies were used for further analysis in the next phase.

In the fourth and final phase, the remaining studies were coded according to a range of variables: authors, year of publication, source journal, area of focus of the journal, study location, type of study, psychological theories used, and outcomes of the studies. Finally, the results of the studies were ranked according to generic and occupation-specific job demands and job resources, job characteristics, socio-demographic data, personal characteristics and a group of miscellaneous. The miscellaneous group consists of determinants that cannot be classified into other categories.

At several points in the procedure, decisions have to be made that are open to discussion. Making those decisions by only one reviewer increases the risk of selection bias. For example concerning the inclusion criteria or the distinction between generic and occupation-specific work demands and resources. Concerning the latter, the distinction between generic and occupation-specific work demands and resources is made by several authors who have used a profession-specific measurement scale, for example, Bakker et al. (2007) concerning teachers and Hakanen et al. (2005) concerning dentists. Therefore, in the present study, we have taken the measurement scale for job-specific work demands and resources from our previous study (Verhoef et al., 2021) as a starting point and the categories in the current study have been adjusted accordingly.

To better compare and interpret the results, two effect sizes were calculated, namely, the frequency effect size (FES) and the intensity effect size (IES) (Onwuegbuzie, 2003; Sandelowski et al., 2007). The FES is used to determine the relative magnitude of the abstracted findings and is calculated by dividing the number of studies that found a result by the total number of studies. The IES represents the impact of a study and is calculated by dividing the number of findings in that study by the total number of results in all studies.

### Quality assessment

As part of a systematic review, a formal assessment of the quality of studies indicates the strength of the evidence on which conclusions are based. This makes it possible to compare studies based on the risk of bias (Whiting et al., 2017). Following the PRISMA statement, the present study established the quality of our identified studies (Page et al., 2021). The quality of the quantitative studies was assessed according to the AXIS procedure (Downes et al., 2016); the quality of the qualitative studies to the CASP procedure for qualitative studies (Long et al., 2020); the quality of the mixed methods studies according to MMAT procedure (Hong et al., 2018); the quality of the meta-analysis according to the 3-MIN procedure (Hussain et al., 2011) and the quality of the systematic review according to the CASP procedure for systematic reviews (Nadelson and Nadelson, 2014).

It is a conventional view that research questions should guide decisions about the design and methodology of a research project (Bryman, 2007). Therefore, it is an essential element of quantitative, qualitative and mixed-method studies (Haynes, 2006). Furthermore, there are various methods to construct a good research question, for example, the Picot criteria (Haynes, 2006) and the Finer criteria (Hulley, 2007).

A hypothesis is a proposed explanation of a phenomenon and is used to answer the research question. As usual, a hypothesis is based on both the theoretical expectation about how things work and on the pre-existing empirical evidence. Finally, hypotheses are tested using the collected data (Haynes, 2006; Hulley, 2007).

Thorough increase and improvement of knowledge are of the utmost importance in any scientific discipline. Therefore, it is also crucial to investigate the quality of a study, and whether a research question, a theoretical foundation, and hypotheses are present in that study. Since the quality assessments used in this literature review, namely the AXIS procedure, the CASP procedure, the MMAT procedure, and the 3-MIN procedure, do not explicitly ask for research questions, theoretical underpinnings and hypotheses, additional attention has been paid to this. Moreover, we investigated whether the outcomes in this study, especially the FES, are influenced by the quality of the studies. We did this by using the Fisher exact test to test the null hypothesis that there is no difference between the FES in the strong studies (*n* = 30) and the moderate studies (*n* = 30) (see Tables 2–7) (Freeman and Julious, 2007).

**TABLE 1:** Results of the systematic quantitative literature review (details of documents included in the data analysis).

No.	Author	Title	Study location	Sample size	Methods used	Psychological theories	Hypotheses (H) or research question (RQ) available	Quality assessment
1	Abrahams et al., 2013	Gender en burnout bij Nederlandse huisartsen.	Netherlands	261	Quantitative	No	RQ	Strong
2	Adam et al., 2018	Potential correlates of burnout among general practitioners and residents in Hungary: The significant role of gender, age, dependent care, and experience.	Hungary	196	Quantitative	No	No	Moderate
3	Adarkwah et al., 2018	Burnout and work satisfaction in general practitioners practicing in rural areas: Results from the HaMEdSi study.	Germany	85	Quantitative	No	No	Strong
4	Ožvačić Adžić et al., 2013	Is burnout in family physicians in Croatia related to interpersonal quality of care?	Croatia	125	Quantitative	No	No	Moderate
5	Akova et al., 2021	Evaluation of the relationship between burnout, depression, anxiety, and stress levels of primary healthcare workers (Center Anatolia).	Turkey	338	Quantitative	No	No	Moderate
6	Anagnostopoulos et al., 2012	Physician burnout and patient satisfaction with consultation in primary healthcare settings: evidence of relationships from a one-with-many design.	Greece	30	Quantitative	The job demands–Resources theory (Demerouti et al., 2001); The conservation of resources theory (Hobfoll, 2002)	RQ	Strong
7	Arigoni et al., 2009	Prevalence of burnout among Swiss cancer clinicians, pediatricians and general practitioners: who are most at risk?	Switzerland	371	Quantitative	No	No	Strong
8	Aygun and Mevsim, 2019	The impact of family physicians’ thoughts on self-efficacy of family physician’s core competencies on burnout syndrome in Ýzmir: A nested case-control study.	Turkey	395	Quantitative	No	RQ	Strong
9	Bakker et al., 2000a	Patient demands, lack of reciprocity, and burnout: A 5−year longitudinal study among general practitioners.	Netherlands	207	Quantitative	The equity theory (Walster et al., 1973); The social exchange theory (Blau, 1964)	H	Moderate
10	Bakker et al., 2001	Burnout contagion among general practitioners.	Netherlands	507	Quantitative	Theory of emotional contagion (Hatfield et al., 1994); Theory of cognitive dissonance (Aronson, 1969)	H	Strong
11	Baptista et al., 2021	Physician burnout in primary care during the COVID-19 pandemic: A cross-sectional study in Portugal.	Portugal	214	Quantitative	No	RQ	Moderate
12	Brown et al., 2019	Personality and burnout among primary care physicians: an international study.	Canada	77	Quantitative	Five-factor theory of personality (Mccrae and Costa, 2008)	RQ	Strong
13	Bugaj et al., 2020	Mental health of postgraduate trainees in primary care: A cross-sectional study.	Germany	211	Quantitative	The control theory of job demands (Karasek, 1979)	H	Moderate
14	Cagan and Gunay, 2015	The job satisfaction and burnout levels of primary care health workers in the province of Malatya in Turkey.	Turkey	186	Quantitative	No	No	Moderate
15	Cheshire et al., 2017	Influences on GP coping and resilience: A qualitative study in primary care.	England	22	Qualitative	No	RQ	Strong
16	Clough et al., 2020	Stressors and protective factors among regional and metropolitan Australian medical doctors: A mixed-methods investigation.	Australia	20/252	Mixed methods	The job demands–Resources theory (Demerouti et al., 2001)	H, RQ	Strong
17	Cooke et al., 2013	A survey of resilience, burnout, and tolerance of uncertainty in Australian general practice registrars.	Australia	128	Quantitative	No	H	Moderate
18	Croxson et al., 2017	GPs perceptions of workload in England: a qualitative interview study.	England	34	Qualitative	No	RQ	Strong
19	Doran et al., 2016	Lost to the NHS: A mixed-methods study of why GPS leave practice early in England.	England	34/143	Mixed methods	No	RQ	Strong
20	Dreher et al., 2019	Prevalence of burnout among German general practitioners: Comparison of physicians working in solo and group practices.	Germany	214	Quantitative	No	RQ	Strong
21	Dusmesnil et al., 2009	Professional burnout of general practitioners in urban areas: Prevalence and determinants.	France	511	Quantitative	No	No	Moderate
22	Eley et al., 2018	Professional resilience in GPs working in areas of socioeconomic deprivation: A qualitative study in primary care.	England	15	Qualitative	No	RQ	Strong
23	Ercolani et al., 2020	Burnout in home palliative care: What is the role of coping strategies?	Italy	207	Quantitative	No	RQ	Strong
24	Ferreira et al., 2021	Burnout and health status differences among primary healthcare professionals in Portugal.	Portugal	1,751	Quantitative	No	RQ	Moderate
25	Galleta-Williams et al., 2020	The importance of teamwork climate for preventing burnout in UK general practices.	England	50	Mixed methods	No	RQ	Strong
26	Gyõrffy et al., 2014	Reproductive health and burnout among female physicians: nationwide, a representative study from Hungary	Hungary	723	Quantitative	No	H, RQ	Strong
27	Hall et al., 2018	Strategies to improve general practitioner wellbeing: Findings from a focus group study.	England	25	Qualitative	No	RQ	Weak
28	Hall et al., 2019	Association of GP wellbeing and burnout with patient safety in UK primary care: a cross-sectional survey.	England	232	Quantitative	No	RQ	Strong
29	van Ham, 2006	De arbeidssatisfactie van de Nederlandse huisarts.	Netherlands	711	Quantitative	The control theory of job demands (Karasek, 1979); The stress theory (Selye, 1950)	RQ	Strong
30	Hirsch and Adarkwah, 2018	The issue of burnout and work satisfaction in younger GPs–A cluster analysis utilizing the HaMEdSi study.	Germany	85	Quantitative	No	RQ	Strong
31	Houkes et al., 2011	Development of burnout over time and the causal order of the three dimensions of burnout among male and female GPs. A three-wave panel study.	Netherlands	212	Quantitative	Gender socialization theory (Benschop, 1996)	No	Strong
32	Houkes et al., 2008	Specific determinants of burnout among male and female general practitioners: A cross−lagged panel analysis.	Netherlands	349	Quantitative	The job demands–Resources theory (Demerouti et al., 2001); The control theory of job demands (Karasek, 1979); The conservation of resources theory (Hobfoll, 2002); Gender socialization theory (Benschop, 1996); Process model of burnout (Leiter, 2018)	H, RQ	Moderate
33	Karakose, 2014	An evaluation of the relationship between general practitioners’ job satisfaction and burnout levels.	Turkey	71	Quantitative	No	RQ	Moderate
34	Kjeldmand and Holmström, 2008	Balint groups as a means to increase job satisfaction and prevent burnout among general practitioners.	Sweden	9	Qualitative	No	RQ	Weak
35	Lamothe et al., 2014	To be or not to be empathic: the combined role of empathic concern and perspective-taking in understanding burnout in general practice.	France	294	Quantitative	No	H	Moderate
36	Lee and Ashforth, 1996	A meta-analytic examination of the correlates of the three dimensions of job burnout.		61	Meta-analysis	The conservation of resources theory (Hobfoll, 2002); Process model of burnout (Leiter, 2018)	RQ	Strong
37	Lovell et al., 2009	May I long experience the joy of healing: professional and personal wellbeing among physicians from a Canadian province.	Canada	165	Qualitative	Ecological model (Bronfenbrenner, 1979)	RQ	Weak
38	Marcelino et al., 2012	Burnout levels among Portuguese family doctors: a nationwide survey.	Portugal	153	Quantitative	No	H, RQ	Moderate
39	Montero-Marin et al., 2015	Mindfulness, resilience, and burnout subtypes in primary care physicians: The possible mediating role of positive and negative affect.	Spain	622	Quantitative	No	H, RQ	Moderate
40	Murray et al., 2016	A systematic review of interventions to improve the psychological wellbeing of general practitioners.		4	Systematic review	No	RQ	Moderate
41	Nørøxe et al., 2018	Mental wellbeing and job satisfaction among general practitioners: A nationwide cross-sectional survey in Denmark.	Denmark	1,697	Quantitative	No	RQ	Strong
42	O’Dea et al., 2017	Prevalence of burnout among Irish general practitioners: a cross-sectional study.	Ireland	683	Quantitative	Physician wellness model (Wallace et al., 2009)	RQ	Moderate
43	Pedersen et al., 2013	Risk of burnout in Danish GPs and exploration of factors associated with development of burnout: A two-wave panel study.	Denmark	216	Quantitative	Process model of burnout (Leiter, 2018)		Moderate
44	Pedersen and Vedsted, 2014	Understanding the inverse care law: A register and survey-based study of patient deprivation and burnout in general practice.	Denmark	601	Quantitative	No	H	Strong
45	Pedersen et al., 2018	Empathy, burnout and the use of gut feeling: A cross-sectional survey of Danish general practitioners.	Denmark	588	Quantitative	Simulation theory of mind-reading (Gallese and Goldman, 1998)	H, RQ	Moderate
46	Pedersen et al., 2020	Influence of patient multimorbidity on GP burnout: a survey and register-based study in Danish general practice.	Denmark	1,676	Quantitative	No	H, RQ	Strong
47	Pedersen et al., 2021	Burnout of intrinsically motivated GPs when exposed to external regulation: A combined panel data survey and cluster randomized field experiment.	Denmark	846	Quantitative	The self-determination theory (Deci et al., 1989)	H, RQ	Strong
48	Putnik and Houkes, 2011	Work-related characteristics, work-home and home-work interference and burnout among primary healthcare physicians: A gender perspective in a Serbian context.	Serbia	373	Quantitative	The job demands–Resources theory (Demerouti et al., 2001)	RQ	Moderate
49	Riley et al., 2018	What are the sources of stress and distress for general practitioners working in England? A qualitative study.	England	47	Qualitative	No	RQ	Moderate
50	Schaufeli et al., 2011	Stability and change in burnout: a 10-year follow-up study among primary care physicians.	Netherlands	567	Quantitative	The selection optimization and compensation (SOC) theory (Baltes and Rudolph, 2012); Motivational theory of life-span development (Heckhausen et al., 2010); Physician-patient cycle model (Williams et al., 2006); Dynamic equilibrium model (Headey and Wearing, 1989); Stability and change model (Ormel and Schaufeli, 1991)	RQ	Moderate
51	Soler et al., 2008	Burnout in European family doctors: the EGPRN study.	England, Greece, Hungary, Italy, Malta, Poland, Spain, Turkey, Sweden, France, Croatia, Bulgaria	1,393	Quantitative	Process model of burnout (Leiter, 2018)	RQ	Moderate
52	Stanetić and Tesanović, 2013	Influence of age and length of service on the level of stress and burnout syndrome.	Serbia	239	Quantitative	No	RQ	Moderate
53	Taris et al., 2005	Job control and burnout across occupations.	Netherlands	562	Quantitative	The control theory of job demands (Karasek, 1979); The conservation of resources theory (Hobfoll, 2002)		Moderate
54	Torppa et al., 2015	Emotionally exhausting factors in general practitioners” work.	Finland	165	Quantitative	No	RQ	Moderate
55	Vedsted et al., 2013	Open access to general practice was associated with burnout among general practitioners.	Denmark	376	Quantitative	No	RQ	Strong
56	Verhoef et al., 2021	Relationship between generic and occupation-specific job demands and resources, negative work-home interference and burnout among GPs.	Netherlands	178	Quantitative	The job demands–Resources theory (Demerouti et al., 2001); The stress theory (Selye, 1950)	H, RQ	Strong
57	Werdecker and Esch, 2021	Burnout, satisfaction and happiness among German general practitioners (GPs): A cross-sectional survey on health resources and stressors.	Germany	549	Quantitative	The job demands–Resources theory (Demerouti et al., 2001); Stress theory of allostatic load (McEwen, 2017)	RQ	Moderate
58	Yuguero et al., 2017a	Association between low empathy and high burnout among primary care physicians and nurses in Lleida, Spain.	Spain	136	Quantitative	No	RQ	Strong
59	Yuguero et al., 2017b	Occupational burnout and empathy influence blood pressure control in primary care physicians.	Spain	267	Quantitative	No	RQ	Moderate
60	Yuguero et al., 2019	A cross-sectional study of the association between empathy and burnout and drug prescribing quality in primary care.	Spain	108	Quantitative	No	H, RQ	Moderate

H, hypothesis; RQ, research question.

**TABLE 2:** Study outcomes job demands.

	Generic job demands	Association with burnout	FES	All studies (*n* = 60)	Strong studies (*n* = 28)	Moderate studies (*n* = 29)	Weak studies (*n* = 3)
1	Burden	+	1.67	1	1	0	0
2	Empathy	+	1.67	1	0	1	0
3	Lack of reciprocity	+	3.33	2	0	2	0
4	Stressful events	+	20	12	12	0	0
5	Workplace incivility	+	1.67	1	0	0	1
6	Complexity of workload	+	5	3	3	0	0
7	Market competition	+	1.67	1	1	0	0
8	Work pressure	+	13.33	9	9	0	0
9	Workload	+	16.67	12	7	5	0
10	Administration	+	8.33	5	4	0	1
**Occupation-specific job demands**							
1	Demanding patients	+	11.67	7	2	4	1
2	Emotional job demands	+	5	3	1	2	0
3	Indirect patient care	+	6.67	4	4	0	0
4	Palliative care	+	1.67	1	0	1	0
5	Patient behavior	+	3.33	2	2	0	0
6	Relationship with insurers increased regulation	+	3.33	2	1	0	1
	Generic job demands	FES_avg_ = 7.33					
	Occupation specific job demands	FES_avg_ = 5.27					

Generic job demands: Fisher exact test of independence, *p* < 0.001; Fisher exact test for difference between strong and moderate studies, *p* < 0.001. Occupation-specific job demands: Fisher exact test of independence, *p* < 0.001; Fisher exact test for difference between strong and moderate studies, *p* = 0.031.

**TABLE 3:** Study outcomes job resources.

	Generic job resources	Association with burnout	FES	All studies (*n* = 60)	Strong studies (*n* = 28)	Moderate studies (*n* = 29)	Weak studies (*n* = 3)
1	Autonomy	−	13.33	8	4	3	1
2	Collaboration	−	5	3	2	0	1
3	Mindfulness	−	6.67	4	0	3	1
4	Motivation	−	5	3	2	1	0
5	Participation	−	3.33	2	2	0	0
6	Personal rewards	−	1.67	1	1	0	0
7	Recovery experience	−	1.67	1	0	1	0
8	Unmet expectations	−	1.67	1	1	0	0
9	Job satisfaction	−	15	11	5	6	0
10	Compulsory daily coffee breaks	−	1.67	1	0	0	1
11	Leisure time	−	3.33	2	2	0	0
12	More administrative staff	−	1.67	1	0	0	1
13	Resilience	−	10	6	5	1	0
**Occupation-specific job resources**							
1	Balint group	−	1.67	1	0	0	1
2	Continuity of care	−	3.33	2	2	0	0
3	Education	−	5	6	3	2	1
4	Skill utilization, including specialization, providing education and sixth sense experience	−	3.33	2	2	0	0
5	Long-term relationship with patients	−	3.33	2	2	0	0
6	Patient care	−	48.33	30	23	5	2
7	Social support from colleagues	−	18.33	11	7	3	1
	Generic job resources	FES_avg_ = 5.38					
	Occupation-specific job resources	FES_avg_ = 11.90					

Generic job resources: Fisher exact test of independence, *p* < 0.001; Fisher exact test for difference between strong and moderate studies, *p* = 0.228. Occupation-specific job resources: Fisher exact test of independence, *p* < 0.001; Fisher exact test for difference between strong and moderate studies, *p* = 0.683.

**TABLE 4:** Study outcomes work characteristics.

	Work characteristics	Association with burnout	FES	All studies (*n* = 60)	Strong studies (*n* = 28)	Moderate studies (*n* = 29)	Weak studies (*n* = 3)
1	Open access	+	3.33	2	2	0	0
2	Practice years	+	6.67	8	1	7	0
3	Practice type	Varying effect	5	3	1	2	0
4	Rurality	+	3.33	2	2	0	0
5	Work hours	+	3.33	2	2	0	0
6	Professional relations with social services and paramedics	Varying effect	3.33	2	2	0	0
7	No of patients per day	+	8.33	8	6	2	0
8	Negative portrayal of the profession	+	3.33	2	2	0	0
9	Aging population	+	1.67	1	1	0	0
10	Changing the relationship between primary care and secondary care	+	3.33	2	2	0	0
11	Illegitimate tasks	+	1.67	1	0	1	0

Fisher exact test of independence, *p* < 0.001. Fisher exact test for difference between strong and moderate studies, *p* = 0.015. Occupation-specific work characteristics. FES_avg_ = 3.94.

**TABLE 5:** Study outcomes personal characteristics [*p*-values are calculated according to Fisher exact (Freeman and Julious, 2007)].

	Personal characteristics	Association with burnout	FES	All studies (*n* = 60)	Strong studies (*n* = 28)	Moderate studies (*n* = 29)	Weak studies (*n* = 3)
1	Depression	+	5	3	0	3	0
2	Anxiety	+	1.67	2	0	2	0
3	Insufficient belief in core competencies	+	1.67	3	2	1	0
4	Neuroticism	+	1.67	1	1	0	0
5	Agreeableness	+	1.67	1	1	0	0
6	Conscientiousness	+	1.67	1	1	0	0
7	Economic status	+	6.67	4	0	3	1
8	Coping	−	6.67	4	2	2	0
9	Positive attitude	−	1.67	1	1	0	0
10	Loneliness	+	1.67	1	0	1	0

FES_avg_ = 3.00. Fisher exact test of independence, *p* = 0.202. Fisher exact test for difference between strong and moderate studies, *p* = 0.129.

**TABLE 6:** Study outcomes socio-demographics [*p*-values are calculated according to Fisher exact (Freeman and Julious, 2007)].

	Socio-demographics	FES	All studies (*n* = 60)	Strong studies (*n* = 28)	Moderate studies (*n* = 29)	Weak studies (*n* = 3)
1	Age	20	14	4	10	0
2	Gender	13.33	12	6	6	0
3	Female	10	6	6	0	0
4	Male	13.33	8	5	3	0
5	Marital status	3.33	2	0	2	0
6	Lack of support partner	1.67	2	2	0	0
7	No of children	1.67	1	0	1	0

FES_avg_ = 9.05. Fisher exact test of independence, *p* = 0.015. Fisher exact test for difference between strong and moderate studies, *p* = 0.015.

**TABLE 7:** Study outcomes miscellaneous category [*p*-values are calculated according to Fisher exact (Freeman and Julious, 2007)].

	Miscellaneous	Association with burnout	FES	All studies (*n* = 60)	Strong studies (*n* = 28)	Moderate studies (*n* = 29)	Weak Studies (*n* = 3)
1	Patient satisfaction	−	1.67	1	1	0	0
2	Burnout among colleagues	+	1.67	1	1	0	0
3	Reproductive disorders (miscarriage/high-risk pregnancy)	+	1.67	1	1	0	0
4	Legal matters	+	5	3	0	3	0
5	Deprived patients	+	1.67	1	1	0	0
6	Patient multimorbidity	+	1.67	1	1	0	0
7	Accreditation	+	1.67	1	1	0	0
8	Patient’s blood pressure control	−	1.67	1	0	1	0
9	Strain-based neg. WHI	+	3.33	2	2	0	0
10	WHI	+	13.33	9	6	2	1

FES_avg_ = 3.34. Fisher exact test of independence, *p* = 0.470. Fisher exact test for difference between strong and moderate studies, *p* = 0.164.

## Results

**FIGURE 1:**
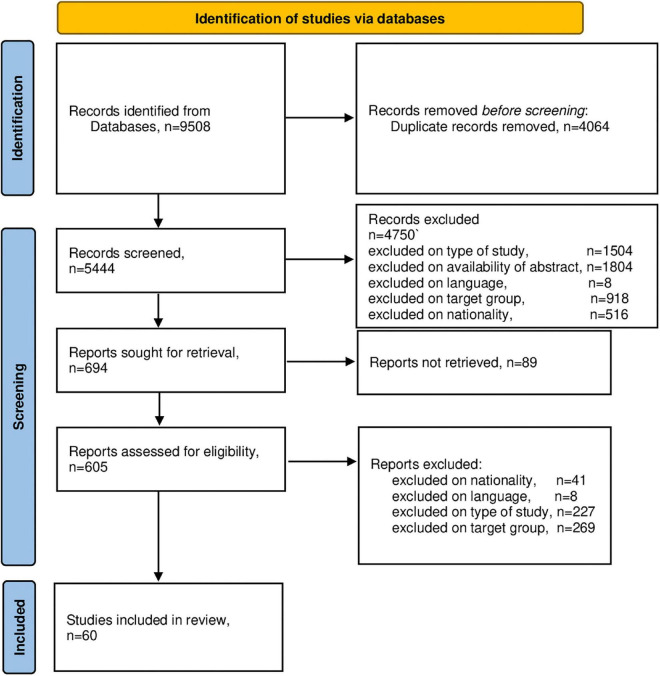
PRISMA 2020 flow diagram. From: Page et al. (2021).

The outcomes of the four screening phases are indicated in the process diagram in Figure 1. The first rough search identified 9,508 studies, of which 60 studies ultimately remained for further analysis. The bibliographical details of these studies (authors and journals) are presented in Table 1. The methodological details of these studies are presented in Table 1. The outcomes of the quality assessments of all studies are presented in Table 1 and are denoted as strong, moderate or weak.

The findings study is explained in detail, including the FES, in Tables 2–8. Table 1 lists the locations of the 60 studies reviewed. Of the 22 different nationalities, the Netherlands and England produced the highest number of studies, namely 9. The psychological theories used for the theoretical underpinning in the studies examined indicate a study’s solidity. Note, that the limited space in a journal can also cause the absence of a theory! Several methodological parameters have been identified and included in Table 1. Of the 24 distinct psychological theories, the COR theory (Hobfoll, 2002) and the JDR theory (Demerouti et al., 2001) have the highest frequencies with 7 (29.1%) and 6 (25%), respectively. Of the 60 studies examined, 65% do not appear to use a supporting psychological theory (see Table 1). Furthermore, of the 60 studies examined, only 10% appear to have formulated and tested a hypothesis and 55% a research question that guides the research.

**TABLE 8:** Intensity effect size (IES) of all studies.

No.	Study	Intensity effect size
1	Abrahams et al., 2013	5.59
2	Adam et al., 2018	1.40
3	Adarkwah et al., 2018	2.45
4	Ožvačić Adžić et al., 2013	2.80
5	Akova et al., 2021	0.70
6	Anagnostopoulos et al., 2012	0.35
7	Arigoni et al., 2009	0.70
8	Aygun and Mevsim, 2019	2.45
9	Bakker et al., 2000a	1.05
10	Bakker et al., 2001	0.35
11	Baptista et al., 2021	1.40
12	Brown et al., 2019	1.05
13	Bugaj et al., 2020	0.35
14	Cagan and Gunay, 2015	0.70
15	Cheshire et al., 2017	4.90
16	Clough et al., 2020	5.59
17	Cooke et al., 2013	1.05
18	Croxson et al., 2017	4.20
19	Doran et al., 2016	2.80
20	Dreher et al., 2019	1.40
21	Dusmesnil et al., 2009	2.10
22	Eley et al., 2018	1.75
23	Ercolani et al., 2020	1.05
24	Ferreira et al., 2021	2.10
25	Galleta-Williams et al., 2020	0.35
26	Gyõrffy et al., 2014	0.35
27	Hall et al., 2018	2.10
28	Hall et al., 2019	1.40
29	van Ham, 2006	0.70
30	Hirsch and Adarkwah, 2018	0.70
31	Houkes et al., 2011	0.70
32	Houkes et al., 2008	2.45
33	Karakose, 2014	1.40
34	Kjeldmand and Holmström, 2008	0.35
35	Lamothe et al., 2014	0.70
36	Lee and Ashforth, 1996	13.99
37	Lovell et al., 2009	3.15
38	Marcelino et al., 2012	1.05
39	Montero-Marin et al., 2015	0.70
40	Murray et al., 2016	0.35
41	Nørøxe et al., 2018	0.70
42	O’Dea et al., 2017	0.70
43	Pedersen et al., 2013	0.35
44	Pedersen and Vedsted, 2014	0.35
45	Pedersen et al., 2018	0.35
46	Pedersen et al., 2020	1.05
47	Pedersen et al., 2021	1.05
48	Putnik and Houkes, 2011	1.40
49	Riley et al., 2018	2.45
50	Schaufeli et al., 2011	0.35
51	Soler et al., 2008	1.05
52	Stanetić and Tesanović, 2013	0.70
53	Taris et al., 2005	0.35
54	Torppa et al., 2015	1.40
55	Vedsted et al., 2013	0.35
56	Verhoef et al., 2021	5.59
57	Werdecker and Esch, 2021	2.10
58	Yuguero et al., 2017a	0.35
59	Yuguero et al., 2017b	0.35
60	Yuguero et al., 2019	0.35

The results of an additional critical appraisal of the quality of the studies are presented as part of Table 1. The impact of a study is represented by its IES and is shown in Table 8 for all 60 studies examined. The mean IES = 1.99 (range 0.35–13.99). This parameter shows that the meta-analysis of Lee and Ashforth (1996) showed an IES = 13.99 and had thus the greatest impact on the current study. In addition, the qualitative study by Clough et al. (2020) (IES = 5.59) has a major impact on the results of the current study.

Finally, a Fisher exact test was conducted to test the null hypothesis that there is no difference between the FES in the strong studies (*n* = 30) and the moderate studies (*n* = 30) (see Tables 2–6) (Freeman and Julious, 2007). Tables 2–7 demonstrate that of the 75 items in total, only two had a significant *p*-value. Those items were Stressful events, Table 2, Job demands, had a *p*-value of 0.0001, and the other item was Patient care, Table 3, Job resources, had a *p*-value of 0.0007.

## Discussion

It is essential to gain a good understanding of the nature and strength of generic work requirements and resources. However, to increase ecological validity, it is also important to identify and further explore occupationally specific work demands and resources (Sundin et al., 2011; Brough and Biggs, 2015).

In the present study, we identified a substantial number of generic and some occupation-specific job requirements and resources for GPs (see Tables 2, 3). To better evaluate the results of a systematic review and the studies used for it, we used two effect measures. The FES of a finding, for example, a job requirement, is considered a measure of the strength of evidence for that finding (Sandelowski et al., 2007). The IES is regarded as a measure of the influence of the study in question on the final result of the ongoing study (see Table 8). For example, a study with an IES of 10 had much more influence on the final result of the ongoing study than a study with an IES of 2. In the current study, we selected 4 studies with relatively high IES, from Table 8, that is, Abrahams et al. (2013) (IES = 5.59), Clough et al. (2020) (IES = 5.59), Croxson et al. (2017) (IES = 4.90), and Cheshire et al. (2017) (IES = 4.90).

The quantitative study by Abrahams et al. (2013) aimed to investigate sex and gender differences in the prevalence and determinants of burnout among 349 Dutch GPs. The authors conclude that there is a relationship between gender and burnout that is mediated by work pressure, social support from the partner and a depressive response pattern. While these are highly recognizable work requirements and resources, they are all generic.

The mixed methods study by Clough et al. (2020) aimed to compare stressors leading to burnout and protective factors among 252 Australian GPs. The authors found 12 stressors (e.g., workload and time management) and nine protective factors (e.g., clinical interest). Except for this last resource, clinical interest, the other stressors and protective factors (resources) mentioned are generic.

The qualitative study by Croxson et al. (2017) aimed to investigate perceptions and attitudes toward workload among 34 UK GPs. The reasons cited for an increased workload are increased needs and expectations of patients, a changed relationship between primary and secondary healthcare and bureaucracy. It is primarily the workload balance within the practice that erodes the resource continuity of care. They, therefore, conclude that management of patient expectations and reduction of bureaucracy should be a high priority. It is the increased expectations of patients and the changed relationship between primary and secondary healthcare that can be regarded as occupation-specific work demands and continuity of care as a profession-specific resource.

Finally, the qualitative study by Cheshire et al. (2017) among 22 British GPs aimed to explore experiences with workplace challenges, stressors and coping. It was experienced as very stressful by the participants to feel bound by the moral implications of good doctors, resulting in anxiety, sleep disorders and stress. As individuals, they felt powerless to do anything about it. Various generic work requirements are mentioned (e.g., administration, workload, complexity of the work) as well as occupation-specific work requirements (e.g., demanding patients). Continuity of care is mentioned as an important occupational resource.

All in all, it is mainly generic work demands and resources that are mentioned in the results of the above four studies and relatively few profession-specific work demands and resources. Moreover, the generic job demands with an FES_avg_ of 7.33 seem more important than the occupation-specific job demands with an FES_avg_ of 5.27 (see Table 2). Further exploration of job-specific job demands and resources among GPs seems highly desirable. The situation is reversed for the resources, the generic resources with an FES_avg_ = 5.38 seem much less important than the profession-specific resources with a much higher FES_avg_ = 11.90 (see Table 3).

The fact that the share of England with nine studies and the Netherlands with nine studies have the largest share in the current study may mean that primary healthcare is changing in both countries.

Finally, Table 1, containing several methodological parameters, raises concerns about the methodological quality of studies. That only 35% of the studies use a supportive psychological theory is low. That only 10% of the studies generate a hypothesis is too low. That 55% of the studies use a directional research question seems average. However, to overcome this concern, we paid additional attention to the presence of the research question, supporting theory and hypotheses in the included studies and whether the quality assessments accounted for these items. Subsequently, we performed Fisher exact tests. From the results, we learned that only two out of 75 *p*-values were significant. We concluded that there was insufficient evidence to reject the null hypothesis. In other words, the quality of the studies did not affect the outcomes of the present study. We consider this finding a strength of this study.

## Conclusion

GPs are independent healthcare professionals in small-scale working-groups outside the hospital. As a medical specialist, the GP has a broad medical knowledge of many diseases and disorders and is the first contact for people with various physical or mental complaints (Verstappen and Hobma, 2002). GPs are considered gatekeepers for hospital access (Van der Zee et al., 2004). The GP can also offer a solution if you have problems at home, school, or work. At least as necessary is GP care for the elderly in an aging society (Verlee et al., 2017; Grol et al., 2022). It is essential that a GP knows their patients and has time for a good conversation (van Ballegooijen et al., 2021). Fewer referrals to hospitals and less medication are also crucial for better health, to which the GP can make an excellent contribution (Gillam, 2022). During the current COVID-19 pandemic, the importance of good primary care has become apparent, as we can also learn from UK experiences (Mitchell et al., 2021). Some GPs vaccinate but also motivate people to get vaccinated. By taking over hospital care by the GP, patients can be discharged earlier and thus reduce the pressure on hospitals (Kloos, 2020; Cals et al., 2021).

If GPs are so important and valuable and have such a high risk of burnout (Bakker et al., 2000b; Soler et al., 2008), why is research into the causes and consequences of burnout among GPs so limited in size? The answer to this question, while partly obscure, is likely to be pluralistic. For example, recruitment problems for participants in research projects in primary healthcare have been reported (Bower et al., 2009; Graffy et al., 2009). But also, understaffed academic institutes for primary healthcare, with too few researchers, contribute to the limited literature (Campbell et al., 2015). Therefore, the urgent call in the current study to stimulate and initiate more research into burnout among GPs requires an ultimate effort that is worth it.

### Limitations

A significant limitation of the current study is that only one researcher conducted the study. Several researchers, at least two, preferably assess the many studies studied in a systematic review. Bias is a systematic error that leads to the acceptance of results and conclusions of a study that can be misleading. Selection bias in a systematic review can be reduced by conducting the study with multiple reviewers (Cooper et al., 2019).

### Future research

Given the results of the present study, it is vital to conduct more qualitative studies to identify and further explore occupation-specific determinants of burnout among GPs. At the same time, this implies that the results of qualitative studies should be generalized by performing quantitative studies. However, given the recruitment constraints and understaffing of academic primary care research institutes, as discussed in the conclusion of the current study, more research will require an extra effort that is well worth it.

## Data availability statement

The original contributions presented in this study are included in the article/supplementary material, further inquiries can be directed to the corresponding author.

## Author contributions

RB supervised this project. NV wrote the main body of the manuscript and carried out selection procedures. Both authors carried out extraction and synthesis procedures, read and approved the final manuscript.
